# Supplementary material for: Conditional chemoconnectomics (cCCTomics) as a strategy for efficient and conditional targeting of chemical transmission
Source: eLife. 2024 Apr 30;12:RP91927. doi: 10.7554/eLife.91927 (PMC11060718; doi:10.7554/eLife.91927)
Supplement: Figure 3—source data 3. [file elife-91927-fig3-data3.zip › Figure 3 source data3 part 1.zip!/Figure 3-Source data 3-part1/1063-13-CRISPResso_Report_8cz_fsdr.zip!/Nucleotide_percentage_quilt.pdf]

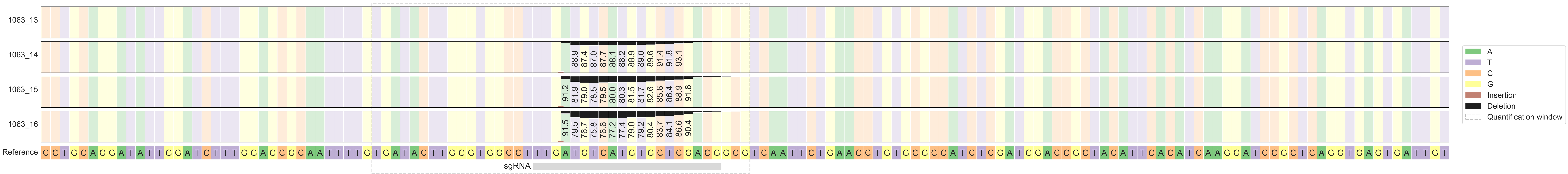

1063_13
1063_14
88.9 87.4 87.0 87.7 88.1 88.2 88.9 89.0 89.6 91.4 91.8 93.1
1063_15
91.2 81.9 79.0 78.5 79.5 80.0 80.3 81.5 81.7 82.6 85.6 86.4 88.9 91.6
1063_16
91.5 79.5 76.7 75.8 76.6 77.2 77.4 79.0 79.2 80.4 83.7 84.1 86.6 90.4
Reference
CCTGCAGGATATTGGATCTTTGGAGCGCAATTTTTGTGATACTTGGGTGGCCTTTGATGTCATGTGCTCGACGGCGTCAATTCTGAACCTGTGCGCCATCTCGATGGACCGCTACATTCACATCAAGGATCCGCTCAGGTGAGTGATTGT
sgRNA
A
T
C
G
Insertion
Deletion
Quantification window
